# Method of Attaching Teeth to Gold Plates by Means of Vulcanite

**Published:** 1891-08

**Authors:** J. Hall Lewis

**Affiliations:** Washington, D. C.


ARTICLE III.

### METHOD OF ATTACHING TEETH TO GOLD PLATES BY MEANS OF VULCANITE.

BY. J. HALL LEWIS, D. D. S., WASHINGTON, D. C.

I have lately noticed several methods of attaching porcelain teeth to gold plates by means of vulcanite. The following I have practiced for the past ten years with much satisfaction. It is equally good for full or partial cases, upper or lower plates, and works best where other methods are least satisfactory as when the porcelain is almost or quite in contact with the gold.

Take an old automatic plugger point, shorten it, file "four sided," then bevel from one angle or corner to a sharp point at the opposite corner, harden, and draw the temper to a full yellow. You then have the ordinary "graver" or "engraver's tool" to fit your automatic mallet. The gold plate being "rimmed" and otherwise ready for the rubber, is placed on the plaster cast and the entire surface of the gold which is to be covered by the aforesaid rubber, is stippled or roughened by means of this "graver" in the mallet, with as heavy a blow as is possible to use without indenting the under surface of the gold. By this means hundreds of little spurs are thrown up from the surface of the gold at various angles to which the rubber attaches itself with remarkable tenacity. Pink rubber should always be used for this purpose, the greater contraction during vulcanization of the other varieties defeating the object desired. If this "stippling" process is thoroughly done, no fear need be entertained of the rubber tearing away from the gold.—*Ohio Journal of Dental Science.*

